# Bonding and Anti-bonding Modes of Plasmon Coupling Effects in TiO_2_-Ag Core-shell Dimers

**DOI:** 10.1038/srep19433

**Published:** 2016-01-14

**Authors:** Quanshui Li, Zhili Zhang

**Affiliations:** 1 Department of Physics, School of Mathematics and Physics, University of Science and Technology Beijing, Beijing, China, 100083; 2 Department of Mechanical, Aerospace and Biomedical Engineering, University of Tennessee, Knoxville, TN, 37996

## Abstract

Bonding and anti-bonding modes of plasmon coupling effects are numerically investigated in TiO_2_-Ag core-shell nano dimers. First, splitting phenomena of the coupled anti-bonding modes are observed under the longitudinal polarization when the distance between the monomers decreases to a certain level. Second, one of the split resonance modes is identified to be formed by the dipole anti-bonding mode of the monomers from charge density distribution patterns. Those split modes have similar redshift behaviors as the coupled dipole bonding modes in the same situations. Furthermore, the intensities of those anti-bonding modes weaken with decreasing distance between the monomers, because of the interaction of the induced dipole moment in the monomers and the charge distribution variation on the facing surfaces of the gap by the coulomb attraction. Other split bands are the higher-order mode (octupole-like or triakontadipole-like), which do not have obvious peak-shift behavior, and the intensities have very little attenuation with decreasing distance. Finally, the coupling of the bonding and anti-bonding modes under the longitudinal polarization is symmetric (bonding).

The coupling of the local surface plasmon resonance (LSPR) in nanostructures is a fundamental plasmonic effect related to many practical applications, such as hot spots for surface enhanced spectroscopy^1^,^2^,^3^, local energy deposition^4^,^5^,^6^, refractive index sensors^7^, and plasmon ruler for the distance sensing^8^,^9^,^10^. Previously dimers^1^,^2^,^4^,^7^,^8^,^9^,^10^,^11^,^12^,^13^,^14^,^15^,^16^,^17^,^18^,^19^,^20^,^21^,^22^,^23^,^24^, trimers^24^,^25^,^26^,^27^, and clusters^26^ formed by pure silver, gold, aluminum, and iron nanoparticles have shown strong plasmon coupling effects, which depend on the symmetry, light polarization, shapes, and configurations. In the dimer configuration, when the light polarization is along the axis of the dimers, the coupled resonance peaks exhibit the redshift behavior as the distances between two monomers decrease; meanwhile the intensities of the plasmonic resonance peaks increase in general^2^,^9^,^10^,^24^,^27^, whereas the coupled resonance peaks show the blueshift behavior as the distances decrease^7^,^9^,^13^,^14^,^15^,^20^ while the intensities of the peaks also decrease^9^ when the polarization is perpendicular to the axis of the dimers. The peak shift behaviors in dimers can be explained by plasmon hybridization modeling^13^,^15^.

The plasmonic hybridization model is analogous to the molecular orbital theory. The plasmon modes of a combined nanostructure can be regarded as resulting from the interactions (hybridization) between the basic plasmon resonances of its elementary components^28^,^29^. In particular, for the simple case (such as dimers and core-shell nanostructures) there are two alignments of the basic plasmonic modes in the hybridization model. One is the symmetric alignment, which results in the bonding (symmetric) mode. The other is the anti-symmetric alignment resulting in the anti-bonding mode. The anti-bonding modes do not exist in the single silver or gold nanoparticles with regular morphologies such as spheres. For the nanoparticles with the irregular shape such as nanostars, the anti-bonding modes may exist, but those modes are weak^30^. From the hybridization model, silver or gold nanoshells are supposed to exhibit the anti-bonding modes and the bonding modes^28^,^29^. However, the anti-bonding modes from the common core-shell nanoparticles, such as silica-gold core-shell nanoparticles^29^, were scarcely identified in experiments and/or simulations. The unapparent or weak anti-bonding resonance bands are due to the inappropriate dispersive dielectric constant of the dielectric cores. It is hard to obtain distinguishable information in the coupling effects from those anti-bonding modes. The recently reported ZrO_2_-Ag core-shell nanoparticles^31^ have the obvious and comparable anti-bonding modes and can be used as the alternative monomers to investigate the coupling of the anti-bonding modes. To date the most reported coupling effects are mainly associated with the interaction of the bonding modes for their diverse applications in the visible range, while the fundamental coupling effects involved in the anti-bonding modes for the complex structures are not yet reported in detail. The coupled bands of the anti-bonding modes are located in the ultraviolet range, so they are expected to be useful in ultraviolet applications.

Here the bonding and anti-bonding modes of the plasmon coupling effects of the TiO_2_-Ag core-shell dimers are investigated. The single TiO_2_-Ag core shell nanostructure has shown two equal intensity dipole plasmon resonance modes, in which one resonance mode is bonding and the other is anti-bonding^32^. The bonding modes are widely tunable from the visible range to the near infrared range with the different core-shell sizes, while the anti-bonding modes are always located at the ultraviolet range with only a slight shift. In this paper, the TiO_2_-Ag core-shell nanoparticles are used as the monomers to generate dimers. The coupling effects from the anti-bonding modes and the bonding modes are investigated by the far field extinction spectra and the charge density distribution patterns. The features of the coupled anti-bonding modes and the coupled bonding modes, the peak-shift behaviors, and their intensity dependence are discussed.

## Methods

The plasmonic properties are calculated by FDTD solutions 8.7 (Lumerical Solutions, Inc.). The refractive indices of silver^33^ and TiO_2_^34^ are from the handbooks edited by Palik. TiO_2_ is a birefringence crystal, however the refractive index can be simplified under the average refractive index approximation by assuming the particles are isotropic^35^,^36^. This approximation is good enough to calculate the far field spectra and the near field patterns of TiO_2_ nanoparticles^35^,^36^. Herein, the complex refractive index can be calculated by *n* = (2*n*_*o+*_*n*_*e*_)/3 and *κ* *=* (2*κ*_*o*_+*κ*_*e*_)/3 respectively. In this study the particles are all in the aqueous medium. The refractive index of water used in FDTD software is set as 1.33. The maximum mesh step for calculating the extinction spectra in FDTD software is set as 0.5 nm, while that for calculating the charge density distribution pattern is set as 0.3 nm. The charge density distribution pattern is calculated from the divergence of the electric field by FDTD software. When the gaps of the monomers in dimers decrease to a very small distance (such as less than 1 nm), the quantum effects (e.g. electron tunneling effect) are obvious^37^,^38^. Here we are concerned about the plasmonic properties of dimers which can be described in the classic electromagnetic theory, so we set the minimum gaps as 4 nm to exclude any quantum effects. In addition, in this study we use the abbreviation to represent the cases of TiO_2_-Ag core-shell nanoparticles; for example, C15S15 indicates that the radius of the Core is 15 nm and the thickness of the Shell is 15 nm for two monomers.

## Results and Discussion

**Figure 1:**
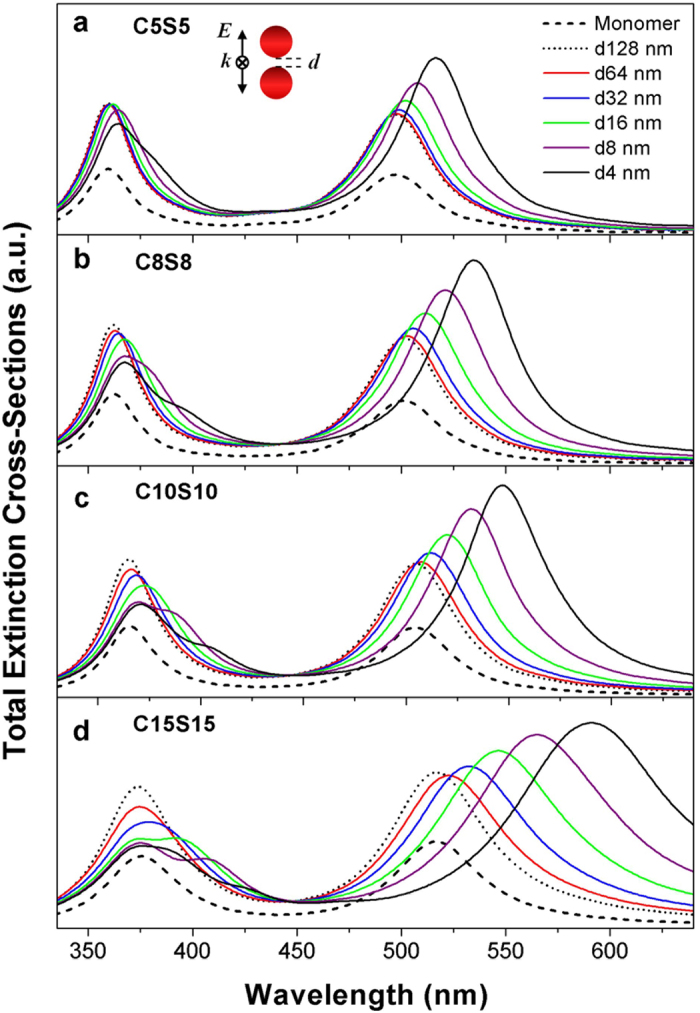
The plasmon coupling effects of dimers under the longitudinal polarization. The dimers are formed by the TiO_2_-Ag core-shell nanoparticles with the different sizes at the same ratio (0.5) of the inner radius to the outer radius. The outer radii are 10 nm (**a**),16 nm (**b**), 20 nm (**c**), and 30 nm (**d**). The distances (*d*) of two monomers are 4 nm, 8 nm, 16 nm, 32 nm, 64 nm, and 128 nm. The extinction spectra of the monomers are also shown. The polarization of light and the direction of propagation are indicated in the figure.

**Figure 2:**
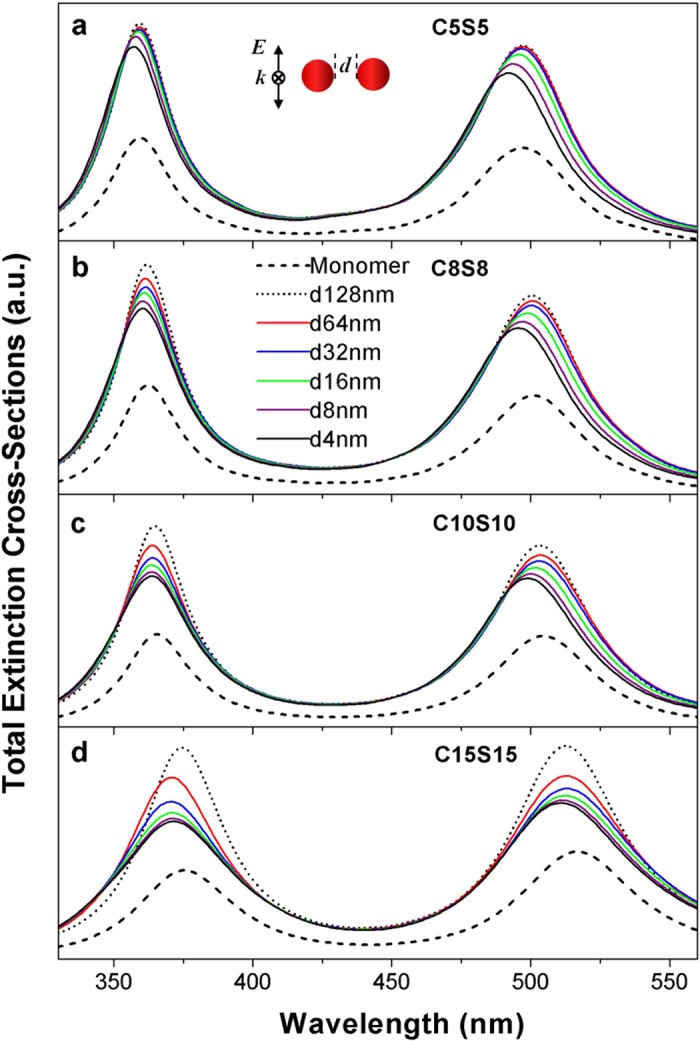
The plasmon coupling effects of dimers under the transverse polarization. The dimers are formed by TiO_2_-Ag core-shell nanoparticles with the different sizes at the same ratio (0.5) of the inner radius to the outer radius. The outer radii are 10 nm (**a**),16 nm (**b**), 20 nm (**c**), and 30 nm (**d**). The distances (*d*) of two monomers are 4 nm, 8 nm, 16 nm, 32 nm, 64 nm, and 128 nm. The extinction spectra of the monomers are also shown. The polarization of light and the direction of propagation are indicated in the figure.

When two core-shell TiO_2_-Ag nanoparticles are approaching each other and form a dimer, the feature of two surface plasmon resonance bands of the single core-shell nanoparticle still remains in the far field extinction spectra as shown in Figs 1 and 2. In general the coupled resonance bands in the extinction spectra can be divided into two ranges; the front one in the short wavelength range is from the coupling of the anti-bonding modes of the monomers, and the other one located at the long wavelength range is from the coupling of the bonding modes. The evolution of the coupled modes is closely associated with the distances of the monomers. When the distances of the monomers are large enough, their interactions are weak. As the monomers approach each other, the interactions of the charges on the surfaces of the metal shells become stronger; in another words, the coupling effects of the plasmon modes become notable. As shown in Figs 1 and 2, the evolution can be found following the decreasing distance. When the polarization of light is parallel to the axis of dimers as sketched in Fig. 1, it can be named as the longitudinal polarization for dimers. The coupled bands from the anti-bonding modes of the monomers exhibit the splitting phenomena with decreasing distance. For the case with the large monomers (C15S15) as shown in Fig. 1(d), the coupled resonance bands can be observed to split with the distances decreasing from 32 nm to 16 nm. The split resonance peaks in Fig. 1 at the longer wavelength side are redshifted with the distances decreasing from 16 nm to 4 nm, while the intensities of those peaks also decrease. The other split resonance peaks don’t exhibit the obvious peak-shift behaviors, and the intensities only attenuate a small amount. Then when the distance is 4 nm, a new split peak around 386 nm emerges between the peak at the 376 nm and the peak at 423 nm like the shoulder of the peak at 376 nm. The splitting phenomena can be easily observed from the coupling of the dimers with the large monomers, while for the coupling of the small monomers, such as C5S5, the splitting phenomena are not observed. Meanwhile, other coupled modes from the bonding modes in the long wavelength range of the spectra are gradually redshifted with the decreasing distance of the monomers and the intensities generally increase, which is consistent with the usual behaviors of the coupling in dimers^2^,^7^,^8^,^9^,^10^,^11^,^12^,^13^,^14^,^15^,^16^,^17^,^18^,^19^,^20^,^21^,^22^,^23^,^24^.

When the polarization of light is perpendicular to the axis of dimers as sketched in Fig. 2, which is named as the transverse polarization for dimers, the coupled bonding modes exhibit slightly blueshift behavior with the decreasing distance of the monomers. This phenomenon is consistent with the usual behaviors in dimers under the transverse polarization^7^,^9^,^13^,^14^,^15^,^20^. The slight blueshift behavior of the coupled modes from the anti-bonding modes of the monomers can be also found, but the splitting phenomena can’t be found. The intensities of the coupled bonding and anti-bonding modes decrease synchronously with decreasing distance.

**Figure 3:**
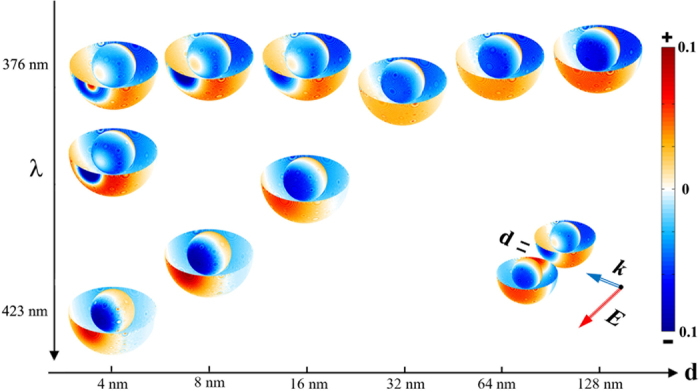
The distribution patterns of the charge densities for the coupled anti-bonding modes under the longitudinal polarization in the 3D drawing. The polarization direction of light (*E*), the direction of propagation (*k*), and the distance (d) between two monomers are indicated in the figure. The monomer is the TiO_2_-Ag core-shell nanoparticle with the outer radius of 30 nm and the inner radius of 15 nm. The charge density distribution patterns are calculated at the resonance wavelengths.

**Figure 4:**
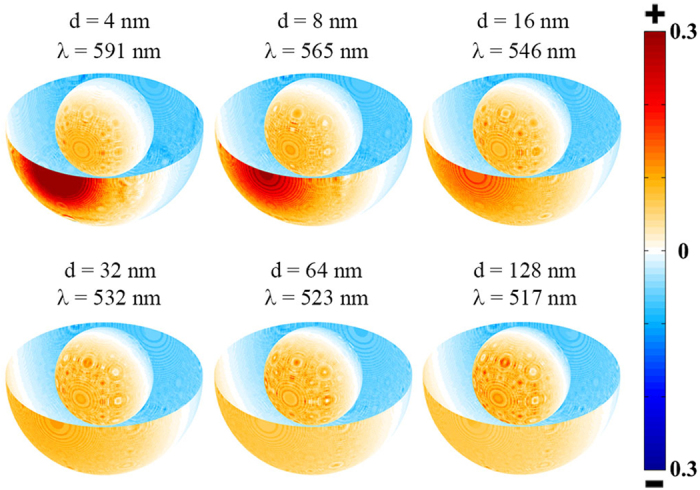
The distribution patterns of the charge densities for the coupled bonding modes under the longitudinal polarization in the 3D drawing. The polarization direction of light (*E*), the direction of propagation (*k*), and the distance (d) between two monomers are indicated in the Fig. 3. The monomer is the TiO_2_-Ag core-shell nanoparticle with the outer radius of 30 nm and the inner radius of 15 nm. The distances and the resonance wavelengths are labeled in the figure.

**Figure 5:**
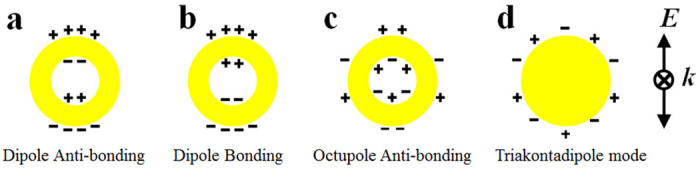
The schematics of the charge distribution patterns of the plasmon modes. The typical dipole anti-bonding mode (**a**), the typical dipole bonding mode (**b**), and the typical octupole anti-bonding mode (**c**) of core-shell nanostructures and that of the typical triakontadipole mode on the metal surface (**d**).

To illustrate the nature of the coupled modes we calculate the charge density distribution patterns as shown in Figs 3 and 4. The coupled anti-bonding modes of C15S15 dimers are displayed in Fig. 3. In figures, One monomer can be used as the representation due to the reflectional symmetry of two monomers. To illustrate the distribution patterns on the inner surfaces, half of the outer surface are removed, which is the reflection of the rest. When the distance is large enough (such as 128 nm), the typical charge distribution of the dipole anti-bonding mode as sketched in Fig. 5(a) emerges on the inner surfaces and the outer surfaces of the metal shells, while as the distances decrease to 16 nm the coupled modes split into two modes. One keeps the typical charge distribution of the dipole anti-bonding mode. Those coupled modes are from two dipole anti-bonding modes. It is worth noting that both sides of the gaps distribute the opposite charges. While the other split modes exhibit more complex behaviors. The new charge distribution firstly emerges on the proximal surfaces of two monomers at the distance of 16 nm, which disturbs the dipole charge distribution. The charge distribution of the split modes will evolve with the decreasing distances. Those coupled modes are not the dipole modes but the higher-order modes. At the distances of 16 nm and 8 nm, the charge distributions on the inner and outer facing surfaces of the semi-shells exhibit the octupole-like feature. When the distance is 4 nm, for the peak at 376 nm, the inner facing surfaces of the semi-shells also exhibit the octupole-like distribution and the outer facing surfaces of the semi-shells exhibit the triakontadipole-like distribution. Then for the mode at 386 nm, the charge distribution patterns on the outer facing surfaces of the semi-shells are octupole-like. The patterns on the inner facing surfaces of the semi-shells tend to be the dipole-like mode, but the top of the inner facing surfaces have the few charge distribution, which is different to the dipole mode. The charge distribution patterns of the octupole anti-bonding modes and triakontadipole modes^39^,^40^ are sketched in Fig. 5(c,d). The charge density distribution patterns of the coupled bonding modes are illustrated in Fig. 4. The coupled modes are coupling from the dipole bonding modes of the monomers as sketched in Fig. 5(b). It is interesting that there are always opposite charges on the surface of the gaps for three types of resonance modes: the dipole anti-bonding mode, the higher-order anti-bonding mode, and the dipole bonding mode. Based on the hybridization model, from the charge distribution of the dimers, it can be concluded that the couplings of the anti-bonding modes of the monomers are both symmetric (bonding) coupling. This conclusion is useful to explain the peak-shift behaviors. Analogous to the molecule orbit hybridization model, the symmetric coupling will be stronger with decreasing distance, which will result in the decreasing energy of the dimers. Therefore, in the coupled spectra the coupled resonance peaks from the dipole anti-bonding and the dipole bonding modes of the monomers exhibit the red shift behaviors under the longitudinal polarization.

When two monomers approach each other, one monomer will feel the coulomb interaction from the charged outer and inner surfaces of another monomer along the polarization direction. The splitting phenomena for the anti-bonding modes can be due to the retardation effects under the influence of another charged outer and inner surfaces. The retardation effects for the large particles result in the higher-order modes^39^. However only the retardation effects cannot generate the higher-order modes for the single core-shell nanoparticle with the outer radius up to 30 nm as shown in Fig. 3 (d = 128 nm). While when the dimers is in the strong coupling, under the influence of another monomers, the higher-order modes emerge. For the monomers with very small sizes, such as C5S5, the retardation effects can be neglected, so the higher-order modes cannot be observed and no splitting phenomena occur.

In the dimer configuration, the intensity dependence of the coupled dipole modes on the distance and the polarization can be qualitatively understood by the interaction of the induced dipole moments in the nearby monomers^20^,^41^,^42^,^43^,^44^,^45^. In the quasistatic approximation, the peak intensities for the plasmon modes mainly composed of the dipole modes are determined by the overall dipole moment^46^. The dipole moments of the monomers can be written as *μ* = *ε*_*m*_*aE*, where *ε*_*m*_ is the medium relative dielectric constant, *a* is the effective polarizability for the monomer, and *E* is the field felt by one monomer, which is the sum of the incident field and the electric field from another dipole moment in the adjacent monomer^41^,^42^,^43^,^44^. The expression for *E* at particle 1 is 
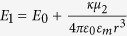
, where *E*_0_ is the incident field, *μ*_2_ is the dipole moment in particle 2, and *r* is the distance of two dipole moments^41^,^42^,^43^,^44^. If the monomers are aligned end to end following the light polarization (*κ* = 2), which is the longitudinal polarization for dimers, the electric field felt by one monomer can be enhanced by the electric field from another dipole moment in the adjacent monomer. Then, with the distances of two monomers decreasing, the enhancement will be stronger so that the dipole moments in each monomer and the dimer will be enhanced, which means the intensities of the resonance peaks also increase with decreasing distance. If the monomers are aligned side by side (*κ* = −1), which is the transverse polarization, the electric field felt by the monomers is attenuated by them both; meanwhile, this attenuation becomes stronger with decreasing distance. Therefore, the dipole moments in each monomer and the dimer will be attenuated, which means the intensities of the resonance peak will also decrease. The intensity dependence of the coupled bonding modes under the longitudinal polarization and the coupled anti-bonding modes under the longitudinal and transverse polarization can be qualitatively explained by the above reason.

The bonding mode in the monomers is mainly from the contribution of the plasmon mode on the outer surface of the metal shell, and the anti-bonding mode is dominated by the plasmon mode on the inner surface^46^. Herein under the end to end alignment, due to the enhanced coulomb attraction of the opposite charges on the nearby surfaces of the gap between the two monomers with decreasing distance, the greater charge densities should distribute on the outer surface, enhancing the bonding modes. Meanwhile, for the coupled dipole anti-bonding mode the charge densities are more concentrated on the facing surfaces of the gap due to the increasing coulomb attraction with the decreasing distance. Then the plasmon modes on the outer surfaces will become significant and suppress the plasmon modes on the inner surfaces by this effect. Then the dipole moment of the dipole anti-bonding modes become weak with the decreasing distances. The intensities of the coupled dipole anti-bonding modes will be attenuated with decreasing distance under longitudinal polarization. As a result, the intensity variations of the coupled dipole bonding modes and the coupled dipole anti-bonding modes have generally opposite behavior under the longitudinal polarization as the monomers approach each other.

Under the longitudinal polarization, the opposite charges distribute on both sides of the gaps for the coupled modes. If the distances of the monomers are very small, the local electric field in the gaps is enhanced, so the dimers can be expected to have application in surface enhanced spectroscopy, especially in the ultraviolet range. In addition, the coupled bonding modes depend strongly on the distances between two monomers, so those peak-shift behaviors can be used to measure the nanoscale distances as a plasmon ruler.

## Conclusions

The dimers formed by TiO_2_-Ag core shell nanoparticles are used to illustrate the plasmon coupling phenomena associated with the anti-bonding modes and the bonding modes. The coupled anti-bonding modes have the splitting phenomena which make the coupled resonance peaks separate with decreasing distance under the longitudinal polarization. The coupled dipole anti-bonding modes and the coupled dipole bonding modes have red shift behavior with decreasing distance, which can be explained by the hybridization model. The intensities of these peaks have opposite behavior due to the interaction of the induced dipole moment in the monomers and the charge distribution variation on the facing surfaces of the gap by the coulomb attraction. Other resonance modes appearing from the splitting phenomena in the coupling of the anti-bonding modes are the higher-order modes (octupole-like or triakontadipole-like), which do not have the obvious peak-shift behavior and have small intensity variation with decreasing distance. Under the longitudinal polarization, the coupling of the anti-bonding modes and the bonding modes is concluded to be symmetrical (bonding). The peak-shift behavior and the intensity dependence for the coupled anti-bonding modes and coupled bonding modes under the transverse polarization are consistent with the usual coupling phenomena of the dimers. The coupled modes have potential application in surface enhanced spectroscopy, especially in the ultraviolet range, due to the enhanced local electric field in the gaps under the longitudinal polarization.

## Additional Information

**How to cite this article**: Li, Q. and Zhang, Z. Bonding and Anti-bonding Modes of Plasmon Coupling Effects in TiO_2_-Ag Core-shell Dimers. *Sci. Rep.* **6**, 19433; doi: 10.1038/srep19433 (2016).
